# Post-COVID-19 Fungal Infection in the Aged Population

**DOI:** 10.3390/vaccines11030555

**Published:** 2023-02-27

**Authors:** Vivek P. Chavda, Toshika Mishra, Sathvika Kamaraj, Swati Punetha, Oishani Sengupta, Yash Joshi, Suneetha Vuppu, Dixa Vaghela, Lalitkumar Vora

**Affiliations:** 1 Department of Pharmaceutics and Pharmaceutical Technology, L.M. College of Pharmacy, Ahmedabad 380009, Gujarat, India; 2 Science, Innovation, and Society Research Lab. 115, Department of Biotechnology, Hexagon (SMV), Vellore Institute of Technology, Vellore 632014, Tamil Nadu, India; 3 Pharmacy Section, L.M. College of Pharmacy, Ahmedabad 380009, Gujarat, India; 4 School of Pharmacy, Queen’s University Belfast, Belfast BT9 7BL, UK

**Keywords:** COVID-19, fungal infections, coinfection, mucormycosis, aspergillosis, invasive candidiasis

## Abstract

Coronavirus disease 2019 (COVID-19) infection is currently a great cause of concern for the healthcare sector around the globe. SARS-CoV-2 is an RNA virus that causes a serious infection that is associated with numerous adverse effects and multiple complications associated with different organs and systems during its pathogenic cycle in humans. Individuals affected by COVID-19, especially elderly populations and immunocompromised people, are greatly vulnerable to opportunistic fungal pathogens. Aspergillosis, invasive candidiasis, and mucormycosis are widespread fungal coinfections in COVID-19 patients. Other fungal infections that are rare but are exhibiting increased incidence in the current scenario include infections caused by *Pneumocystis jirovecii*, *Histoplasma* sp., *Cryptococcus* sp., etc. By producing virulent spores, these pathogens increase the severity of the disease and increase the morbidity and fatality rates in COVID-19 patients globally. These infections generally occur in patients recovering from COVID-19 infection, resulting in rehospitalization. Older and immunocompromised individuals are at higher risk of developing opportunistic fungal infections. This review focuses on understanding the opportunistic fungal infections prevalent in COVID-19 patients, especially elderly people. We have also highlighted the important preventive methods, diagnostic approaches, and prophylactic measures for fungal infections.

## 1. Introduction

Fungal infections mainly comprise toxin-lethal dose-level opportunistic infections in humans, which include aflatoxin and ochratoxin from Aspergillus, acetaldehyde from Candida, and Rhizonin A from Rhizopus. Fungal infections when occurring as a coinfection in patients were reported to increase their morbidity and mortality [1]. SARS-CoV-2 has spread alarmingly through communities and infected people of all ages, ethnic backgrounds, and genders, but its severity is greater in aged populations [2]. Fungal infections occurring with COVID-19 can enhance the pathogenicity and virulence of COVID-19 infection and may even cause death. Effective laboratory testing is essential to determine whether a person has a COVID-19 infection, a fungal infection, or both. A severe COVID-19 disease that increases the risk of bacterial and fungal infections is associated with decreased expression of CD4 interferon-gamma, lower counts of CD4 and CD8 cells, and an increase in proinflammatory cytokines such as IL-1, IL-6, and tumor necrosis alpha, leading to higher chances of mucormycosis [3].

A study conducted to understand COVID-19 and fungal coinfections reported that approximately 54.6% of COVID-19 sufferers died from contracting secondary fungal infections. Thus, fungal coinfections increase the death rates in COVID-19 patients [1].

Recent studies have shown that bacterial coinfection in COVID-19-positive patients causes a twofold increase in the fatality rate, whereas fungal infections increase the fatality rate by 4-fold [4].

Coinfection is more prevalent in people over the age of 50 years than in younger populations. According to a UK study on patients with COVID-19 and fungal infections, the age range of 55 to 81 years had the highest percentage of coinfected patients [5]. Similarly, another study in the United Arab Emirates determined the median age of patients with coinfections and reported it as 49.3 ± 12.5 years [6]. Additionally, a study conducted in Spain also found that the COVID-19 patients who had fungal infections were mostly 62 years old [7].

## 2. Development of Fungal Infections during COVID-19

The common fungal infections prevalent in patients are aspergillosis, invasive candidiasis, mucormycosis, respiratory alveoli infection, and other epidermal infections. Viral pneumonia is known to cause concomitant infections or secondary infections. One-fourth of H1N1 patients during the 2009 pandemic had a bacterial or fungal infection, based on a retrospective study. A similar pattern is observed in the case of COVID-19 patients [8]. Studies have documented more than six million fatalities worldwide and over 500 million cases of invasive fungal infections in COVID-19 patients [9]. Asia, America, Europe, Africa, and Australia reported 49.7, 23.2, 19.8, 6.6, and 0.5% rates, respectively, for the development of opportunistic fungal secondary infection in COVID-19 patients [1]

Currently, a huge number of problems involving secondary health infections, idiopathic infections, iatrogenic infections, superinfections, and coinfections are prominent among COVID-19-positive individuals [10]. Numerous risk factors make people prone to fungal infections, such as respiratory difficulties, skin infections, black eye fungal infection, immunosuppression, the requisite for supplemental oxygen, monoclonal antibodies, steroid therapy, etc. Patients with COVID-19 experience adverse complications that are closely related to opportunistic fungal infections, triggering the development of new terms for coinfections, such as COVID-19-associated mucormycosis (CAM), COVID-19-associated pulmonary aspergillosis (CAPA), and COVID-19-associated candidiasis (CAC) [10].

**Figure 1:**
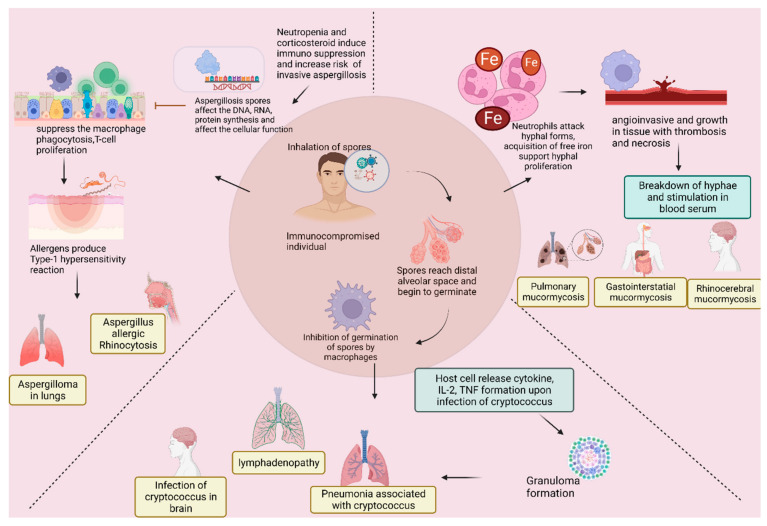
Mechanism of the pathogenesis of invasive aspergillosis, mucormycosis, and cryptococcus fungal infections and their complications (Created using Biorender.com).

Some of the quintessential fungal infections seen in COVID-19-infected individuals include aspergillosis, candidiasis, cryptococcosis, and mucormycosis. These infections are caused by *Aspergillus* spp. *Candida auris, Cryptococcus neoformans*, and fungi belonging to the Mucorales order, respectively [11]. The mechanism of pathogenesis of invasive aspergillosis, mucormycosis, and cryptococcus fungal infections is presented in Figure 1.

### 2.1. Root Cause of Coinfection

Scientists and medical experts have affirmed that the ideal circumstances for fungi to infect people with COVID-19 are low oxygen conditions brought on by the patient’s hypoxemia, high glucose levels brought on by diabetes (a well-recognized risk element) or steroid-induced hyperglycemia, decreased white blood cell phagocytic activity carried on by the suppression of the immune response of the host by the virus and/or steroid treatment, and an acid-rich environment brought on by diabetic ketoacidosis and elevated ferritin levels due to elevated iron levels [12]. A cohort investigation in Wuhan, China, looked at critical patients in acute respiratory distress. The results showed that patients with longer hospital stays (more than 2 weeks), particularly those who were admitted to the hospital’s ICU and required ventilator support, had an increased risk of developing a fungal coinfection [12].

### 2.2. Diabetes—The Main Inducer of Opportunistic Fungal Infection

Additionally, it has been demonstrated that patients infected with SARS-CoV-2 have hyperglycemia, which almost doubles the chances of superinfection and coinfection. Mucormycosis and candidiasis exhibited an especially high correlation with the disease, even though diabetes raises the chance for many infections. It has been reported that in 101 mucormycosis cases linked to COVID-19, 80% of infected patients had diabetes mellitus (DM) before infection [13]. Studies report a greater prevalence of fungal infections such as mucormycosis in India because the country has the second largest population with diabetes, which is an important risk factor [14].

The possible mechanisms that resulted in enhanced COVID-19 morbidity and fatality by diabetes include reduction in viral clearance, depletion in T-cell function, elevation in a cytokine storm, and prevention of the cascade of immune responses. Patients with COVID-19 experience multiorgan damage as a result of hyperglycemia, which exacerbates the cytokine storm and disturbs endothelial cells. People who have foot ulcers and chronic diabetes are particularly vulnerable to fungal infection because damage to skin tissue provides a site for the entry of fungal pathogens [15].

Patients with diabetes frequently have clinically uncontrolled diabetes and elevated blood glucose levels, which are ideal for the rapid growth of filamentous structures. These structures initially attach to the periphery of blood vessels and then penetrate them, forming clogging entirely in a short period and leading to vast areas of ischemic necrosis [16].

## 3. Fungal Infections in COVID-19 Patients

Fungal infections by opportunistic fungal species have become a serious cause of concern, especially for COVID-positive patients, as they lead to increased hospitalization and fatality rates. For a patient in the ICU, the increase in the number of hospitalization days in the ICU and the incubation time corresponds to an increased risk of opportunistic fungal infections. Consequently, this not only increases the period of stay in the hospital but also the death rates in patients [4]. The incidences of various fungal infections have increased rapidly during the COVID-19 pandemic. However, mucormycosis, invasive aspergillosis, and invasive candidiasis are the most prevalent secondary infections occurring with COVID-19 [14].

### 3.1. Aspergillosis

Aspergillus species are widespread fungi that enter the host system by inhaling airborne spores and can result in fatal infections, especially in hosts who are immunocompromised. Aspergillus species are frequently isolated from indoor environments such as hospitals as well as soil, plant residues, and enclosed spaces. Aspergillus, primarily *A. fumigatus,* causes lung diseases that result in a variety of clinical manifestations in the lungs. Invasive pulmonary aspergillosis (IPA) is a serious condition that causes an increase in the death rate of severely immunosuppressed individuals. In the absence of conventional risk factors, critically ill patients without malignancies can indeed develop IPA. Locally invasive chronic necrotizing aspergillosis (CNA) is a lung infection caused by *Aspergillus* spp. CNAs are primarily found in mildly immunocompromised patients or those with chronic lung disease [17]. *Aspergillus* spp. can also cause noninvasive lung diseases such as aspergilloma and allergic bronchopulmonary aspergillosis (ABPA) [17].

Conidia production marks the beginning of Aspergillus’ infectious life cycle. Conidia are ubiquitous and airborne in both indoor and outdoor settings. These airborne conidia are the main source of infection in humans, and after being inhaled, they are then deposited in the bronchioles or alveolar spaces [18]. Epithelial cells or alveolar macrophages, the two main resident phagocytic cells of the lung, come into contact with conidia that are not cleared by mucociliary clearance in healthy individuals. Alveolar macrophages are primarily involved in phagocytosing and killing Aspergillus conidia and initiating pro-inflammatory responses that recruit neutrophils (polymorphonuclear cells [PMN]) to sites of infection [19]. Conidia that survive elimination by macrophages and germinate are targeted for neutrophil infiltration, which destroys the hyphae. The chance of contracting invasive aspergillosis (IA) is basically due to the breakdown in host defenses, combined with fungal characteristics that allow *A. fumigatus* to survive and grow in the lungs [19].

Immunosuppression, resulting from neutropenia and induced by corticosteroids, is the primary host immunodeficiency that contributes to the increased risk of IA. Cytotoxic medications such as cyclophosphamide [20], which are prescribed to patients with hematologic disorders or transplant recipients, are frequently used to treat persistent neutropenia, the main risk factor for developing IA. Cyclophosphamide, a DNA alkylating agent, binds to DNA, disrupts cell replication, and depletes circulating leukocytes, including neutrophils.

Many nonneutropenic patients undergo corticosteroid therapy. For instance, allogeneic transplant patients generally receive corticosteroids for the prevention or treatment of graft-versus-host disease. Patients with IA are predisposed to the disease, but the pathology varies greatly. IA is nonvascular invasive in these patients, with limited fungal development, pyogenic granulomatous infiltrates, tissue necrosis, and excessive inflammation [19].

Conidia of Aspergillus are so prevalent in the environment that breathing them in is a common occurrence. It is estimated that an average person can inhale up to 200 conidia per day. In a population prone to IA, pulmonary mucosal defenses are rendered inefficient, leading to fungal colonization and proliferation [19,21]. Although new antifungal medications have been developed, the treatment of IPA is still difficult, with high death rates. As soon as clinical suspicion of IPA exists, treatment should be taken into consideration, and monitoring should be continued. Since many years ago, amphotericin B has been the first-choice IPA, with doses of 1–1.5 mg/kg/day. However, amphotericin B can cause serious adverse events such as nephrotoxicity, electrolyte imbalance, and hypersensitivity. Both avoiding patient hospitalization and using high-efficiency particulate filtration (HEPA) with or without laminar flow ventilation are beneficial approaches. It was discovered through a meta-analysis that itraconazole can shield neutropenic patients from fungal infections. Chemoprevention studies with other antifungal agents (e.g., voriconazole, caspofungin, micafungin) in high-risk patients are currently underway [17].

Chronic necrotizing aspergillosis (semi-invasive or subacute invasive aspergillosis) occurs in the lung parenchyma as a result of local invasion by an Aspergillus species, most commonly *A. fumigatus* [19,22]. CNA is typically linked with chronic lung conditions such as COPD, previous pulmonary tuberculosis, thoracic surgery, radiation therapy, pneumoconiosis, cystic fibrosis, pulmonary infarction, or (less commonly) sarcoidosis. A meta-analysis revealed that itraconazole can shield neutropenic patients from fungal infections [17,19].

### 3.2. Invasive Candidiasis

Invasive candidiasis is an opportunistic infection caused by Candida species in immunocompromised patients. Studies have revealed that over 90% of infections are caused by the species *C. albicans*, *C. glabrata*, *C. krusei*, *C. tropicalis,* and *C. parapsilosis*. The yeast genus Candida includes commensal microbes in humans that are prevalent on the skin and mucous lining of the oral cavity and gastrointestinal and genitourinary tracts. They have the ability to cause superficial and invasive infections in people with low immunity [23]. The incidence of this infection has increased rapidly across the globe [24]. It is a common infection in seriously ill patients and is linked to significant morbidity and mortality [25]. Invasive candidiasis incidences have rapidly increased in recent years, affecting individuals all over the globe [26].

The infection is noted to have a complex pathogenesis that is primarily influenced by colonization, alteration of physical barriers, and deficiencies in phagocytes and/or immunological responses that regulate invasion at the mucosal site [27]. *Candida* spp. are generally commensal organisms present in the gut and skin. However, disruption of the mucosal lining in addition to the weakened immune response by the host can facilitate the transformation to opportunism that further introduces important virulence factors resulting in the infection. The progression of invasive Candida infections is primarily caused by three factors. This includes prolonged and repeated use of broad-spectrum antibiotics that raise the number of *Candida* spp. in the gut by depleting the commensal microbiota, which plays an important role in inhibiting the overgrowth of Candida. Other factors include a rupture of the gastrointestinal and cutaneous lining and iatrogenic immunosuppression due to immunosuppressive therapy or neutropenia induced by chemotherapy [28]. The host’s immune response can be rendered ineffective against *Candida* spp., as they can avoid complement attacks by two approaches. They can either express complement regulators on the surface (such as Factor H, C4BP, plasminogen, etc.) or cause protease-dependent degradation of complement proteins [29]. In an immunocompromised individual, superficial candidiasis can easily transition into a severe invasive form, consequently accumulating in vital organs and thus causing disseminated candidiasis. In the case of COVID-19 patients, the drugs available further suppress the already suppressed immune response [30]. As one of the most frequently detected pathogens, Candida is found to affect approximately 8 to 10% of patients in the intensive care unit (ICU). Several studies have shown an exponential rise in candidemia cases [11].

Invasive yeast infections are among the serious COVID-19 complications that are being recognized. Clinical factors such as prolonged ICU stays, central venous catheters, and the use of broad-spectrum antibiotics may have a substantial impact on COVID-19 patients acquiring the infection [11]. However, the exact mechanism of pathogenesis in COVID-19-positive patients is yet to be understood. The absence of adequate information on the possible risk factors that make COVID-19-positive people prone to infection, along with this knowledge gap, could result in the misdiagnosis of secondary candidiasis in COVID-19 patients. The important determinants that contribute to the development of candidiasis in COVID-19 patients include ICU admission, antibiotics, corticosteroids, weak immunity, deficiency of iron and zinc, etc. [31]. Furthermore, studies have also revealed that age is an independent risk factor for mortality in invasive candidiasis patients, with individuals over the age of 65 years found to be highly susceptible to the infection. The rising incidence of infection in the immunocompromised elderly population in addition to the development of treatment resistance may be contributing factors to the growing problem of invasive Candida infections. Alterations in the pharmacokinetics and pharmacodynamics of older individuals, particularly frail and severely ill individuals, are the reason for their experience of adverse drug effects. Organ dysfunction, decreased homeostatic control, comorbidities, and polypharmacy may make this situation even more challenging [31]. Thus, this infection is a great cause of concern for COVID-19 patients because it elevates mortality rates [32].

To treat elderly patients, various antifungal medications can be utilized. However, echinocandins are found to be the most efficient option with minimal adverse effects and drug‒drug interactions compared to other prophylactic regimens, with the exception of their effectiveness against the resistant strains *C. glabrata* and *C. krusei* [33].

### 3.3. Mucormycosis

Mucormycosis is a severe fungal infection caused by Mucorales and is known to infect immunocompromised individuals globally. There are six families in the order Mucorales that cause superficial and deep infections. The majority of mucormycosis cases are caused by the family Mucoraceae, and *Rhizopus oryzae* (*Rhizopus arrhizus*) is presently the most common infectious agent [34]. The Mucoraceae family also includes *Absidiacorymbifera*, *Rhizopus microsporus var. rhizopodiformis*, *Apophysomyces elegans*, *Mucor* spp., and *Rhizomucorpusillus*, all of which cause a wide range of infections [34]. An increase in cases of mucormycosis due to infection with *Cunninghamella* spp. has also been reported [35].

Mucormycosis is a severe opportunistic fungal infection that results in increased hospitalization and mortality in vulnerable population groups, such as immunocompromised and elderly individuals over the age of 65 years. The prevalence of mucormycosis has rapidly increased throughout the world, especially among people with COVID-19 [36]. Studies reveal that during the second wave of COVID-19 infection, India reported the maximum incidences of mucormycosis because the country has the maximum cases of diabetes mellitus, hypertension, and other metabolic deficiencies in aged populations compared to the world [37]. These patients primarily experienced dyspnea and cough, and the most typical laboratory test results were elevated levels of C-reactive protein (CRP), PCT, D-dimer, ferritin, and lymphopenia [38].

Normal hosts’ mononuclear and polymorphonuclear phagocytes kill Mucorales by producing oxidative metabolites and the cationic peptide defensin. Clinical evidence suggests that phagocytic cells are the primary defense mechanism of the host against infection. For instance, neutropenic patients have a greater chance of developing it. Furthermore, patients with poorly functioning phagocytic cells are more likely to develop mucormycosis. Increased susceptibility to infection in patients with elevated serum iron is a significant clinical feature that has recently been identified. It has been acknowledged for 20 years that patients on treatment with the iron chelator deferoxamine exhibit a significant increase in cases of invasive mucormycosis. It is now known that deferoxamine does not encourage Mucor infection through iron chelation [34].

Mucormycosis is distinguished by the nearly uniform presence of extensive vascular infiltration, leading to vascular thrombosis and tissue necrosis. The ability of the organism to propagate hematogenously from the initial site of infection to other target organs is connected to this vascular invasion. As a result, a crucial part of an organism’s pathogenic strategy may involve harming and invading the endothelial cells that line blood vessels [34].

The prevalence of mucormycosis is lower than that of other opportunistic fungal infections, such as those caused by Candida and Aspergillus. According to population-based studies, there are 1.7 cases of infection for every million people annually, which corresponds to approximately 500 cases in the United States annually. In serial autopsies, the frequency ranges from one to five cases per 10,000 autopsies, 10 to 50 times lower prevalence than invasive Candida or Aspergillus infections [34]. Furthermore, its incidence is said to be 2–3% in high-risk patients, including individuals who have undergone allogeneic bone marrow transplantation. Mucormycosis can be segmented into at least six different clinical categories based on its clinical signs and intervention at specific anatomical sites: rhinocerebral, pulmonary, cutaneous, gastrointestinal, disseminated, and miscellaneous [34,35]. With one-third to half of all mucormycosis cases, the rhinocerebral type continues to be the most prevalent illness. Approximately 70% of rhinocerebral (sometimes called craniofacial) incidences are reported in diabetic patients with ketoacidosis. In rare cases, rhinocerebral mucormycosis develops in people who have solid organ transplants or who have persistent neutropenia. Recently, there has been an increase in rhinocerebral disease in patients undergoing hematopoietic stem cell transplantation. These cases are primarily linked to the use of steroids in the treatment of graft-versus-host disease [39].

#### 3.3.1. Development of Mucormycosis in COVID-19:

COVID-19 infection fosters an environment that is favorable to mucormycosis and becomes more prevalent in patients who are battling or recovering from COVID-19 [40]. Humans can contract two different types of mucormycosis infection.

Superficial and Visceral

A specific type of mucormycosis called superficial mucormycosis frequently occurs when fungal spores enter the host through a skin break. Surgical procedures, severe burns, or other types of skin trauma are common triggers for this infection. Visceral mucormycosis is more likely to develop in young children than in adults. Infants who are less than one month old who were born prematurely, who had surgery and received antibiotics, who were born underweight, or who are taking medications that weaken the body’s defenses against infection are in the risk category [41].

Superficial: The skin, fingernails, and external ears all demonstrate superficial forms of mucormycosis infection. Superficial mucormycosis is the third most prevalent clinical manifestation. An opportunistic fungus of the phylum Glomeromycota causes the emerging fungal infection known as cutaneous mucormycosis. Clinical implications of cutaneous mucormycosis are nonspecific. It is frequently observed in diabetic patients and immunocompromised individuals. It is crucial to identify the fungus as quickly as possible to begin antifungal treatment. To increase survival in cases of cutaneous mucormycosis, treatment requires multidisciplinary strategic techniques [42]. Comprehensive surgical excision, antifungal therapy, correction of the underlying metabolic or immunological status that is compromised, and management of any simultaneous infections should all be part of this procedure [42].

Visceral: Pulmonary, gastrointestinal, and rhino-cerebral infections are manifestations of the visceral type [43]. By consuming infectious agents in foods such as fermented milk and dried bread products, one can develop gastrointestinal mucormycosis. Due to the nonspecific nature of the disease’s presentation and the need for prompt endoscopic biopsy analysis, gastrointestinal mucormycosis prognosis is typically delayed. The most common risk factors are massive gastrointestinal hemorrhage, which is caused by the fungus invading blood vessels and bowel walls, which can lead to bowel perforation, peritonitis, sepsis, and other conditions [44].

Local and Disseminated

Skin lesions that tend to stay localized and can subsequently extend over months and years are assumed to be the localized form of mucormycosis. One form that spreads quickly and is considered fatal is disseminated mucormycosis. Nine percent of cases of mucormycosis are caused by disseminated mucormycosis, which has a high mortality rate [45].

Localized: The localized form of mucormycosis is rare and occurs as gastrointestinal, endocarditis, osteoarticular, or isolated cerebral infections [22].

Dissemination: Mucormycosis can be disseminated from one organ to another hematogenously. The lung is most frequently associated with dissemination. Furthermore, the digestive system, burns, and severe cutaneous lesions all contribute to the progression of the disease. To diagnose mucormycosis earlier than usual, a metastatic skin lesion is used as a significant marker. Disseminated mucormycosis will always be fatal if not properly treated [44].

#### 3.3.2. Types of Mucormycosis

Bloodstream-mediated further fungal hyphal dissemination results in a number of infections, such as rhinocerebral mucormycosis, pulmonary mucormycosis, and gastrointestinal mucormycosis. The distinctive feature of rhinocerebral mucormycosis is caused by tissue necrosis by angioinvasion and subsequent thrombosis [46]. Typically, these appear as dark and necrotic eschars. Upon inhalation, the fungus enters the paranasal sinuses, from which it may eventually spread to the sphenoid sinus, palate, and cavernous sinus [47]. Consequently, people with the infection may suffer from blurred vision, sinusitis, orbital inflammation, facial pain or numbness, headaches, proptosis, ophthalmoplegia (weakness of eye muscle), or even periorbital cellulitis [46].

Gastrointestinal (GI) mucormycosis, a rare, frequently opportunistic, potentially fatal angio-invasive infection, affects 4 to 7% of all cases [48]. The stomach, colon, and ileum are the three organs where GI mucormycosis is most prevalent. The most typical clinical manifestations of GI mucormycosis include generalized abdominal distention and pain in addition to nausea and vomiting [47]. A biopsy of the suspected area is typically used to make the diagnosis during surgery or endoscopy. Early diagnosis, effective and timely antifungal therapy, and surgical debridement are necessary for successful management [48].

A significant proportion of immunosuppressed individuals have a rare fungal infection known as pulmonary mucormycosis (PM) [49]. Pulmonary mucormycosis is characterized by fever, hemoptysis, and tissue infarction [50]. Over the past few years, early diagnosis, surgical excision, and newer antifungal medications have all enhanced the prognosis and consequences of this infection [50]. Early diagnosis and risk factor management, appropriate surgical removal, and prior to treating any of these coinfections, proper antifungal medication must be administered.

Hospitalized COVID-19 patients, particularly the aged population, as well as individuals with severe symptoms who require mechanical ventilation, are provided corticosteroids to relieve some of their symptoms. However, it is well known that steroids can impair a patient’s immune system, elevate blood sugar, and generally raise levels of clotting factors and fibrinogen. This circumstance gives pathogens a chance to infect the host while eluding the human immune system. Recent studies have shown an increasing number of cases of mucormycosis in COVID-19 patients who have been hospitalized or recovered. An invasive, potentially deadly fungal infection that primarily affects people with weakened immune systems and damages the nose, eyes and brain. Patients with type 2 diabetes, autoimmune diseases, iatrogenic suppression of the immune system, or hematologic cancers and patients with organ transplants with blood glucose levels above 220 mg/dL are particularly susceptible to this condition [8].

### 3.4. Miscellaneous Fungal Infections

Numerous studies have been performed on COVID-19 patients and associated fungal infections. These studies reveal that various other rare or less frequent fungal species, such as *Histoplasma* spp., *Cryptococcus* spp., and *Pneumocystis jirovecii,* are also rapidly increasing and causing superinfections. The most efficient diagnostic methods for the detection of fungal infections include a sputum test, RT‒PCR, and CT scan of the chest [3]. The elderly population is highly vulnerable to such secondary infections due to reduced immunity. The risk of invasive fungal infections increases many-fold by infection with SARS-CoV-2 [51].

*Pneumocystis jirovecii*: *Pneumocystis jirovecii* has been identified in severe cases of COVID-19, causing coinfection with *Pneumocystis pneumonia*. Those who were immunosuppressed and infected with SARS-CoV-2 displayed substantial lymphopenia and altered lymphocyte functions, explicating the high detection rates of the fungus [3]. This secondary infection is rare but critical in worsening patients’ conditions and ultimately leading to their deaths [52]. Pneumonia caused by *P. jirovecii* is also reported to lead to rehospitalization of patients recovering from COVID-19 [52].

*Cryptococcus* spp.: *Cryptococcus species,* especially *Cryptococcus neoformans,* are reported to cause cryptococcal disease in immunocompromised patients. Studies have revealed that this opportunistic species causes coinfections in COVID-19 patients with an increase in the risk of mortality within 30 days of infection. Therefore, it is crucial to detect the infection at an earlier stage to reduce the mortality rate [3]. At the age of 65 years and older, respiratory conditions and corticosteroid use with a daily dose of corticosteroids equivalent to 5 mg of prednisone are all significant risk factors for serious infection by *C.* neoformans in tocilizumab-treated patients [53]. It is also reported to cause fatal disseminated infections in immunosuppressed individuals. Studies suggest that this fungus can cause serious cryptococcal meningitis posttreatment of COVID-19 with the drug dexamethasone in susceptible groups [54].

*Histoplasma* spp.: Disseminated histoplasmosis is reported to develop in immunocompromised individuals. Coinfection of COVID-positive patients with *Histoplasma* spp. increases the severity of symptoms such as respiratory failure. To lower the risk of death from such infections, effective and proactive diagnosis and treatment are required [55]. Those with severe COVID-19 and those receiving large dosages of steroids and immunosuppressants are among the groups at risk for developing histoplasmosis [56]. *Histoplasma capsulatum* is known to cause severe acute disseminated histoplasmosis in individuals with no history of infection by this species but develops it post-infection by SARS-CoV-2 [51].

Table 1 discusses the recent studies on coinfection by fungal pathogens, which are rapidly rising in COVID-positive patients.

## 4. Impact of Fungal Infections on Recovery

In 2021, several reports from all over the world brought forth another catastrophe, i.e., the growing number of fungal coinfections in COVID-19 patients. Patients over the age of 65 who had previously been infected with COVID-19 and were now undergoing a recovery period have a higher risk of getting infected with fungus because their already weakened immune systems deteriorated with the treatment strategies used against the virus. This causes the immune system to be less effective against fungal assault [1]. In addition, severe COVID-19 causes an increase in proinflammatory mediators, such as IL-1, IL-6, and tumor necrosis factor-alpha (TNF-α), less CD4 interferon-gamma (INF-γ) expression, and fewer CD4 and CD8 cells, which enhance susceptibility to both bacterial and fungal infections inside the body [3]. Basile et al. presented how in 23 countries, there were more COVID-19-infected patients who had fungal infections in the age group of 50 and above than in the younger age group [1]. A study conducted in India by Munipati et al. discovered that the predominant population that had post-COVID fungal infections belonged to the age bracket of 41–60 years, followed by the population between 61–71 years [72].

These COVID-19-associated fungal infections have been more prevalent, and more time is required for the recovery of an aged patient who had just overcome the COVID-19 infection. COVID-19-associated pulmonary aspergillosis (CAPA) is more severe because the clinical symptoms and radiological results are very similar to those of severe COVID-19, making detection of the disease difficult. The pathological blood tests used for diagnosis of the fungi lacked sensitivity because of the invasion of the airway *by Aspergillus* and the removal of *Aspergillus* galactomannan (GM) from the systemic circulation by neutrophils in patients who were nonneutropenic [61]. This brought about a clinical picture of the fungal infection that differed from the primarily angio-invasive invasive pulmonary aspergillosis (IPA), as was seen in patients with neutropenia; CAPA is frequently limited to causing airway invasive growth for numerous days before it finally becomes an angio-invasive phenomenon. However, the death rate increases to over 80% when CAPA becomes angio-invasive and releases positive serum GM, even though systemic antifungal therapy is being given to control it. [73]. Bronchoscopies were sparsely carried out, particularly in the early days of the pandemic, due to the high risk of COVID-19 transmission in this case. Prior detection is possible through testing of bronchoalveolar lavage (BAL) for CAPA. The results of CAPA have been alarmingly poor; studies estimate that CAPA contributed independently to an increase in mortality rates of more than 40% [61].

One of the main causes of invasive yeast infections in COVID-19 patients is prolonged hospitalization, followed by the use of central venous catheters and broad-spectrum antibiotics. Several studies have revealed a high prevalence of Candida infections in COVID-19 patients, making Candida and several of its species potential pathogens in such patients. Candida species are most predominantly reported on the skin’s mucosal surfaces, as well as the respiratory, urinary, and gastrointestinal tracts [28]. Organisms of the genus *Candida,* with *Candida albicans* being the major species, are the most common types of pathogenic yeast recovered from ICUs, affecting 6% and 10% of the patients admitted. *C. albicans* and *C. auris* infections were related to significantly high comorbidities and death rates. In the case of neutropenic patients and patients being treated with azole therapy, infection by other less common species, such as *C. glabrata*, *C. dubliniensis*, *C. parapsilosis*, *C. tropicalis* and *Pichia kudriavzevii,* which is known as *C. kruseias*, was especially observed and reported [23,74]. Invasive candidiasis fatalities among ICU patients are between 19% and 40%, but they can reach up to 70%. In addition, other yeast pathogens, such as Rhodotorula and Saccharomyces, can also cause infections in these patients. [74]. Thus, *Candida* infections fatally increase the severity of infection among elderly COVID-19 patients and dangerously jeopardize their recovery from the disease [75].

Studies have demonstrated that the primary causes of COVID-19-associated mucormycosis are an extremely weak immune system, prolonged and heavy steroid use, and broad-spectrum antibiotics used during the treatment of seriously ill SARS-CoV-2 patients [76]. Sinonasal mucormycosis infection is an acute invasive fungal infection, and especially in immunocompromised patients, it is disseminated faster to the other organs [72]. Rhinocerebral mucormycosis is very dangerous, as it causes the death of the patient within a week if the doctor is unable to correctly diagnose the infection. The hemorrhage and tissue destruction (tissue necrosis) caused by the fungus are extremely severe and sometimes spread to the gastrointestinal tract, skin, and lungs, which further causes a delay in the complete recovery of the patient. Due to a complete lack of circulating neutrophils brought on by hematologic malignancies such as leukemia and lymphoma, patients also experience severe immunocompromised conditions [77]. The effects of mucormycosis were more severe in patients with diabetes, and in some cases, the disease’s effects were so serious that the doctors had to perform surgical removal of the patient’s diseased jaw and eyes to stop the spread of the illness throughout the body. It is crucial to correctly identify opportunistic fungal infection at the outset. The diagnosis will depend on early identification of risk factors, clinical symptoms, and radiological abnormalities, as well as confirmation by culture and biopsy until better molecular diagnostic techniques and biomarkers are made available.

There were certain other fungal diseases, such as valley fever (coccidioidomycosis), blastomycosis, and histoplasmosis, that could cause symptoms such as cough, fever, swelling face, and shortness of breath, which are comparable with those of COVID-19 and bacterial pneumonia. These soil-dwelling fungi can be transmitted between people through inhalation, as they are air-borne pathogens that can hinder the recovery of patients [74].

Thus, it is important that these fungal infections are correctly diagnosed with suitable methods to lessen their impacts and prevent them from spreading so that aged patients can recover faster and in a healthier way.

## 5. Prevention, Diagnosis, and Treatment

### 5.1. Prevention

Recent research has demonstrated that healthy diets, raw food nutrients, natural products, and micronutrients, including conventional herbal treatments such as Chinese medicine, can reduce hospitalization and the severity of the pulmonary impact of COVID-19 [78,79]. Predicting severe and immediate respiratory distress following SARS-CoV-2 infection may be controlled with a basic understanding of people’s nutritional status and oxidative scavenging capacity [80,81,82,83,84].

Effective methods for avoiding and reducing the prevalence of SARS-CoV-2 include the practice of social distancing and the creation of appropriate space arrangements. Physical distance can be an inefficient approach in a crowded setting. A method based on simulation integrates physical-distancing pedestrian dynamics with an infection judgment framework to determine the effect of spatial management on the fine-scale transmission of disease [85].

A complete shutdown might be considered the most effective way to stop and control the COVID-19 outbreak in public areas, but it results in enormous financial losses. Additionally, some public spaces must remain open to support fundamental social functions and meet bare necessities. Social distancing, sanitation, and disinfection practices in addition to precautionary measures are some of the important recommended approaches to control the spread of infection [86].

The current requirement is to modify diet and lifestyle practices to lessen the effects of complications associated with infection [87,88,89,90].

### 5.2. Diagnosis

A population of immunocompromised people who are at high risk during the COVID-19 pandemic is particularly vulnerable to invasive fungal infections. The majority of conventional regular diagnostic techniques, such as histopathology, are still regarded as the gold standards; however, due to their low sensitivity, there is an increased need for the establishment of newer techniques for identifying pathogens with a fungal nature. Several novel molecular and serologic techniques have been developed, and many of them are under clinical evaluation to test their sensitivity, such as that of the Galactomannan Antigen Test for Aspergillosis [91]. PCR and added molecular approaches, such as matrix-assisted laser desorption ionization (MALDI) and fluorescence in situ hybridization (FISH), have demonstrated reliable results in clinical trials but must be standardized prior to being introduced for clinical applications [92]. Here, the diagnostic approaches for the main three invasive fungal diseases, aspergillosis, candidiasis, and mucormycosis, are tabulated in Table 2.

### 5.3. Treatment

The complex medical circumstances of elderly COVID-19-positive individuals and the inapt collection of their clinical samples have caused the majority of opportunistic fungal infections, primarily mucormycosis, aspergillosis, and candidiasis, in this group of patients to be misdiagnosed [105]. This has caused serious repercussions during their COVID-19 recovery period as well.

The European Confederation of Medical Mycology (ECMM) and mycosis study group education research consortium developed different approaches and management of mucormycosis [38]. Prompt and complete surgical treatment should be taken first, and systemic antifungal drugs such as isavuconazole and proconazole should be given as first-line management. The treatment of SARS-CoV-2-associated pulmonary aspergillosis (CAPA) is generally divided into two categories: allergic aspergillosis and invasive aspergillosis. Voticonazole, posaconazole, isavuconazole, itraconazole and lipid amphotericin B formulations can be given in the treatment of invasive aspergillosis. During treatment with antifungal drugs, if the patient develops aspergilloma, then surgery may be required [106]. A novel echinocandin and biafungin is currently under preclinical studies and has shown early promise in mouse models as a once-in-week treatment for the prevention of IA [107]. *C. auris* is usually treated with echinocandins, as it is rarely susceptible to azoles or amphotericin B. During treatment, therapeutic drug monitoring should be required to optimize the efficacy and limit the toxicity of azoles [108]. The combination therapy of trimethoprim and sulfamethoxazole has been given as a treatment for pneumocystis pneumonia infection. Instead, corticosteroids can also be used for moderate to severe pneumonia [109]. Early detection of infection and recommended treatment with antifungal drugs may be helpful in the prevention of disease.

In a recent small, retrospective study, patients with diabetes who received combination LFAB-caspofungin therapy had significantly better outcomes for rhino-orbital-cerebral mucormycosis than those who received polyene monotherapy [110]. One of the preclinical and confined retrospective clinical findings indicated that combining LFAB and echinocandin therapy might indeed enhance mucormycosis survival.

- Patients receiving protracted treatment for chronic pulmonary aspergillosis showed similar outcomes in a different retrospective study. When compared to patients who received voriconazole, patients who received isavuconazole experienced markedly fewer side effects [111].
- In a recent cohort study, Shoham et al. found that 32% of 32 patients with hematological malignancies and respiratory infections responded to LAmB as the initial treatment for mucositis [110].
- The two most recent preclinical studies have looked at the effectiveness of the posaconazole combination for murine mucormycosis. According to that study, posaconazole plus LAmB did not extend survival in mucormycosis-infected neutropenic or DKA mice over LAmB monotherapy [110].

Proper early diagnosis and suitable antifungal treatment strategies thus become necessary to treat fungal infections at the earliest. Additionally, since the management of fungal infection in such patients costs a considerable amount of wealth to both the public and private sectors per annum, appropriate planning must be done while laying out the treatment strategies, which should not only be the most effective means of healing but also the most economical one. This is crucial for low- and middle-income countries, where a lack of resources has a significant impact on the availability and effectiveness of therapeutic approaches. [62].

In Table 3, the presently available treatment options for the invasive fungal infections of aspergillosis, candidiasis, and mucormycosis are summarized.

## 6. Conclusions

A high proportion of epidemiological as well as observational studies and case reports from two years after the pandemic outbreak continue to illustrate the clinical implications of COVID-19 that can frequently be involved in secondary respiratory fungal infections. The chance of contracting and aggravating existing opportunistic fungal infections seems to be ramped up by the combined effect of diabetes and increased corticosteroid utilization to treat COVID-19 infection. The manifestation of different fungal coinfections in COVID-19 patients appears to be influenced by mechanical ventilation, catheterization, and immunosuppressive treatments. As a result, when caring for COVID-19 patients, doctors and other healthcare providers should be aware of the potential risks and the likelihood of secondary infections.

The only ways to alleviate the situation of this devastating disease are early and prompt diagnosis, recovery from predisposing factors, and early intervention with surgical excision and therapeutic drugs. Therapeutic agents such as corticosteroids, immunosuppressants, and broad-spectrum antibiotics must be used with caution, both in terms of dosage and duration.

## Figures and Tables

**Table 1:** Studies on opportunistic fungal infections in COVID-positive patients.

Pathogen	Study Type with Reference	Population	Risk Factors	Comments
*Aspergillus* spp.	Retrospective analysis [57]	The study was conducted on 70 patients of a tertiary hospital in North India.	Diabetes mellitus	The probability of developing the condition increases drastically due to comorbidities such as Diabetes mellitus. Aspergillus causes angioinvasive infections. The diagnostic approaches followed in the study include a clinical and radiological examination. The frequent symptoms in patients were headache, nasal congestion, palatal ulcer, and loss of vision with facial pain and swelling.
	Prospective study [58]	The study was performed on 10 patients with subacute invasive pulmonary aspergillosis at All India Institute of Medical Sciences (AIIMS), New Delhi, India	Steroid administration Diabetes	The main symptom observed in patients was cough and preceded by hemoptysis. In this study, clinical, radiological, and microbiological examination tests were performed to identify the pathogen. Serum galactomannan detection and molecular techniques such as PCR were also employed for the diagnosis of the infection.
	Retrospective study [59]	The study was conducted on patients from two tertiary hospitals in the United Kingdom.	Hypertension	The mortality rate of COVID-19-associated pulmonary aspergillosis is much higher compared to normal COVID-19 patients. The diagnosis mainly depends on the study which includes clinical examination, RT‒PCR, matrix-assisted laser desorption/ionization time-of-flight mass spectrometry (MALDI-TOF)
*Candida* spp.	Retrospective analysis [60]	The data was obtained from people infected globally.	Diabetes Obesity Hypertension Old age Corticosteroid and steroid administration Prolonged ICU admission Usage of broad-spectrum antibiotics Kidney diseases Mechanical ventilation	Laboratory, imaging, and clinical examinations are the important diagnostic approaches followed in the study. Prolonged antifungal usage can lead to bloodstream infections.
	Prospective research [61]	The study considered populations from low, middle, and high-income countries.	Administration of antibiotics Central venous catheters Corticosteroid administration ICU admission	The common diagnostic approaches include clinical and radiological techniques. In some cases, invasive diagnostic techniques such as histology and autopsies are used. Interleukin inhibitors or tocilizumab could also be one of the causes of fungal infections caused by *Candida* spp., *Histoplasma* spp., and Pneumocystis jirovecii. Fluconazole resistance is observed in a majority of C. auris strains.
Species of order Mucorales	Retrospective analysis [13]	The study was performed on 101 patients, where 82 were from India and 19 were from other parts of the world.	Diabetes Overuse of corticosteroids	The data was retrieved from RT‒PCR-based diagnosis. Higher incidences in males. Rhino-orbital type is the second most prevalent form of the condition, preceded by nose and sinus mucormycosis.
	Prospective study [62]	This study is generalized for the complete human population.	Suppressed immunity Presence of comorbidities	The study discussed various molecular assays and techniques such as CRISPR‒Cas 9, MALDI-TOF, sequencing analysis, PCR-based approaches, etc., for accurate and reliable detection of the causative species. Globally, the rhino-orbital-cerebral type of mucormycosis has the maximum prevalence. Many common antifungal agents are rendered ineffective for the treatment of mucormycosis. Several ongoing studies are targeting different resistance mechanisms of the pathogen to design novel effective, treatment options.
	Retrospective cross-sectional study [40]	All patients with COVID-19-associated mucormycosis in the Assiut University Hospital were participants in the study.	Males Smokers Hypertension Diabetes Prolong inflammation Misuse of steroids.	The study discussed the detection techniques such as laboratory-based techniques, radiology, histopathology, and surgery.
	Retrospective study [63]	The majority of work was on Indian populations. However, reports of COVID-19-associated mucormycosis from other parts of the world are also included.	Obesity Formation of cytokine storm Use of contaminated nonsterile clinical objects	The diagnostic techniques discussed include radiographic and histopathological studies.
	Retrospective analysis [64]	The study focused on the complete human population.	Hyperglycemia Suppressed immunity Transplantation Ketoacidosis Neutropenia Diabetes.	The diagnosis techniques in practice are clinical examinations (such as ocular examination), imaging techniques (such as Computerized Tomography scan, Magnetic Resonance Imaging, and endoscopy), histopathological studies, and microscopic examination using culturing and molecular detection using qPCR and RLFP. The MALDI-TOF technique is used for confirmatory diagnosis.
*Pneumocystis jirovecii*	Prospective study [65]	The study aimed to discuss Pneumocystis pneumonia with respect to the general human population.	Enhanced inflammatory response Immuno-deficiency HIV infection Corticosteroid administration	Detection of Pneumocystis jirovecii is difficult specifically in the case of COVID-19 patients because the methods that offer desired sensitivity involves invasive sampling methods.
	Retrospective study [66]	The study focused on a 52-year-old male patient from Romania.	COVID-19 or HIV Administration of immunosuppressants Malignancies Immunodeficiencies	The clinical and radiological examination can yield false negative results. Bronchoscopy and bone marrow biopsy can provide a diagnosis of the condition when the imaging and laboratory tests prove to be inefficient.
	Retrospective study [67]	The study reports the case of a 71 year old male patient from Iran.	Cancer	The diagnosis requires invasive sample collection techniques to provide sensitivity to the examination, therefore not recommended for serious patients. Commonly used techniques for diagnosis include qPCR, β-D-glucan test, imaging, and serological tests.
*Cryptococcus* spp.	Retrospective study [68]	The study focused on a male patient in his early 1970s from the USA.	Autoimmune diseases Diabetes mellitus Cancer	Microscopic analysis of blood culture can be used to detect the infection of cryptococcus. The other approaches include histological diagnosis using chemical or immunofluorescence staining techniques, and RTqPCR.
	Retrospective study [69]	The study aimed to analyze cryptococcosis in COVID-19 patients in the global population.	Chronic disorders	Imaging techniques (such a CT scans, and MRI), serological tests, clinical examination, and bronchoscopy are some of the diagnostic techniques employed for the detection of *Cryptococcus* spp. Cryptococcosis was associated with enhanced fatality among COVID-patients.
	Retrospective analysis [70]	The study was performed on a 46-year-old male patient in South Korea.	Alteration of immune response to COVID-19 infection	The detection of Pulmonary cryptococcosis was performed by histopathological studies of the biopsy samples obtained from the lesions of the lung of the patient.
*Histoplasma* spp.	Retrospective analysis [55]	A 61-year-old male patient from Texas with a history of obesity, hypertension, diabetes, and hypothyroidism.	Suppressed immune response	CT-guided percutaneous needle biopsy of the infected lung tissue followed by a histopathological study of the biopsy sample was performed for diagnosis.
	Retrospective analysis [71]	The study focused on a kidney transplant recipient from Argentina having COVID-19 and disseminated histoplasmosis.	Kidney transplantation	The effective detection method for histoplasmosis is a microscopic examination of biopsy samples. The diagnostic techniques include molecular PCR approach, CT scan, serological examination, bronchoalveolar lavage, tissue biopsy, and urine analysis.
	Retrospective analysis [56]	The study was aimed at a 65-year-old female patient from India with a comorbid condition of nonalcoholic steatohepatitis associated chronic liver disease.	Old age Suppressed immune response	Lung biopsy and serological examination are important diagnostic approaches.

**Table 2:** Diagnostic approaches for invasive fungal infections.

Serial	Diagnostic Technique	Principle	Advantage	Disadvantage	Comment	Reference
1.	Potassium hydroxide test	Nonfungal components are completely dissolved by the potassium hydroxide (KOH) solution and fungal hyphae and yeast cells are visualized under a microscope.	Use of very simple instruments to perform a preliminary experiment and detect the fungus. Easy to perform with minimal requirement of technical assistance. Economical	The strains of the fungus in the present specimen are very difficult to distinguish. Requires experience to efficiently remove the cellular material from the background to facilitate easy distinction.	This quick test is frequently used to diagnose superficial fungal infections and is frequently carried out in pathology or microbiology labs.	[93,94]
2.	Biopsy	Infected body tissue is obtained from the patient to check for the infection. It is often performed in combinations such as CT scan guided or video-assisted methods to surgically obtain the sample in cases of deep tissues.	The sensitivity and specificity of this method are 100%	Very invasive. It can be harmful to people who are vulnerable to invasive infections (For instance, in the scenario of a deep tissue biopsy). Requires trained practitioners to perform it.	Gold standard diagnostic technique	[92,95,96]
3.	Antibody testing	Includes immunodiffusion, complement fixation, and enzyme immunoassay with immunodiffusion detecting antibodies precipitating with *Histoplasma* M and H antigens.	A very specific test can be conducted in laboratories with mediocre set-ups. Provides positive results in cases where samples are difficult to obtain. Positive results reduce the requirement of culturing the samples. Less invasive.	Due to antibody cross-reactivity with other viruses, there are high chances of receiving false positive results in antibody immunoassays. Low sensitivity and specificity.	This nonculture-based immunodiagnostic test method is most preferred in cases to establish the infection of endemic mycoses.	[94,95,97]
4.	Polymerase Chain Reaction (PCR)	In vitro replication of DNA or RNA with the help of polymerase enzyme. It provides accurate molecular identification of the fungal pathogen.	Accurate identification of the species of fungus involved. Does not require live cells for the process.	Most of the PCR fungal assays are not standardized. Expensive approach as it requires organism-specific probes for detection.	Although there are numerous commercial and internal tests available, there are no PCR assays for Candida that have been FDA-cleared.	[38,95]
5.	Matrix-assisted Laser Desorption Ionization (MALDI)	An analytical technique in which the organism is identified based on its peptide mass fingerprint.	Very fast and does not take more than five minutes to correctly identify microorganisms from isolated colonies. Can identify the strains directly from blood, cerebrospinal fluid, and urine. Efficient in identifying filamentous fungi (95% accuracy for Aspergillus).	Can only be found in sophisticated laboratories because of the high initial setup costs. Accuracy is low for two pathogens that have similar mass spectra.	MALDI-TOF in conjugation with an antimicrobial stewardship team reduced organism identification time, thereby improving outcomes for patients and antimicrobial agent selection. Efficient in identifying filamentous fungi and *Candida* spp.	[38,92,98,99]
6.	Fluorescence in situ hybridization (FISH)	An approach employs fluorescent probes to identify specific regions on the genomes of microbial pathogens in human samples, which could be detected by fluorescence microscopes.	Highly accurate detection of the pathogen. Easy detection directly from blood smear, without the requirement of amplification of nucleic acids.	False positive and negative results Incomplete hybridization Nonspecific binding Expensive	Can be used even in frozen tissue sections.	[92,99,100]
7.	Beta-D-glucan (BDG) testing	Factor G has been identified to be a component that interacts with BDG from fungi.	In comparison to more focused assays for candidiasis, BDG has a significant benefit for detection.	The absence of effective sensitivity and specificity for a conclusive diagnosis with a positive test result is one drawback of β-D-glucan analysis.	Beta-d-glucan is an enticing antigen since it is prevalent in a wide variety of fungal organisms, including *Candida* spp., *Aspergillus* spp., and Pneumocystis jirovecii.	[101]
8.	Serological testing for Histoplasma detection	These tests are based on antigen-antibody interactions.	Leading diagnostic test for detection of *Histoplasma* spp. Noninvasive, widely accessible, and high sensitivity, and specificity.	The major limitation is the cross-reactivity of antibodies during the testing. Occurrence of false positives.	The antigen testing method is more efficient than antibody testing due to greater sensitivity and specificity The detection of antigens in urine has greater sensitivity than serum in all types of histoplasmoses. A combination of antigen and antibody examination can facilitate improved sensitivity in diagnosis.	[102]
9.	BAL Galactomannan testing	This type of Galactomannan testing is done on bronchoalveolar lavage (BAL). In this technique, an instrument is passed through the oral or nasal passage and a fluid is released into a region of the lung that collects the fungi and microbes present in the lungs and analyzes them using ELISA.	It is considered the most accurate test for the detection of Invasive Aspergillosis. The test has high sensitivity and specificity for Invasive Aspergillosis. ∙	As the test is invasive, it is not employed for regular screening but only on patients with the risk of Invasive Aspergillosis.	The tests present considerable heterogeneity. It is useful in differentiating people with a high probability of developing Invasive Aspergillosis and people with a lower possibility of developing it.	[103,104]

**Table 3:** Treatment measures for invasive fungal infections.

Fungal Infection	Available Treatments	Comment	Clinical Data	Reference
Aspergillosis	Voriconazole with the dose range of 6 mg/kg IV every 12 h per day, followed by 4 mg/kg every 12 h for 6–12 weeks. For Per oral administration 200 mg at every 12 h	Voriconazole is available in tablet, IV, and suspension formulations for the treatment of invasive aspergillosis. Reported to cause liver and gastrointestinal abnormalities. Voriconazole has 96% oral bioavailability and causes high CNS penetration.	In phase II and III clinical trials, voriconazole showed exemplary clinical efficacy in human subjects. A randomized trial involving 144 patients. In a comparative study in which IV amphotericin B deoxycholate and voriconazole were given to different patients; after 12 weeks the survival rate was 70.8% in the voriconazole group and 57.9% in the amphotericin group.	[112,113,114,115]
	Isavuconazole in the dose range of 200 mg every 8 h per 6 doses, then 200 mg IV or oral daily for 6–12 weeks.	Isavuconazole, a potent second-generation triazole available in capsule and IV formulations in the treatment of IA. Reduced efficacy as both Voriconazole and Isavuconazole are substrates for CYP3A4 enzyme in the liver. It reduces drug‒drug interaction compared to other triazoles	An interventional open-label trial involving 149 patients investigated that Isavuconazole was found to be effective and generally well tolerated in international phase III clinical trials for the treatment of invasive aspergillosis fungal infection.	[112,113,116,117] NCT00634049
	Posaconazole was administered in the dose range in IV 300 mg twice daily and then 300 mg once daily for 6–12 weeks.	Posaconazole is available in slowly released tablets, oral suspension, and IV formulation to treat refractory IA. If obtainable, only to be utilized as a second therapeutic option.	The FDA has authorized the use of posaconazole (Noxafil, Schering Corporation, Kenilworth, NJ) in immunocompromised patients as a preventative treatment against invasive Aspergillus and Candida fungi infections. An open-labeled trial stated the use of posaconazole as a salvage therapy for IA patients who were intolerant of previous antifungal treatment. The overall success rate was 42% for posaconazole.	[112,118,119,120]
	Liposomal amphotericin B should be administered at a dose of 3–5 mg/kg per day.	IV amphotericin B binds to ergosterol in a fungal membrane by forming pores leading to cell death. L-AmB has shown a positive correlation between in vivo susceptibility and clinical outcomes in IA patients Patients with COVID-19 who have renal dysfunction ought to avoid it. Side effects of LAmB such as diarrhea, nausea, vomiting, electrolyte imbalance, and nephrotoxicity.	In a pooled analysis trial investigating L-AmB for the treatment of IA and other fungal infections in immunocompromised individuals, the response observed approximately 51% of cases of fungal infections. L-AmB remains sensitive against most Aspergillus spp, and increasing rates of elevated MICs >2 have been reported for Aspergillus fumigatus.	[61,112,113,121,122]
Invasive Candidiasis	Echinocandins such as anidulafungin, caspofungin, and micafungin	Echinocandins, the newest class of antifungals, exhibit fungicidal effects in Candida species. It is act by inhibition of β-D-glucan synthase. The combination of drugs meets with severe drug resistance from the fungal isolates obtained from the COVID-19 patients. A novel echinocandin rezafungin with a dose once every week has produced activity against Candida species.	According to a recent meta-analysis of randomized clinical trials in C/IC, echinocandins were not inferior to other relevant antifungals in terms of efficacy but had a greater safety profile. Rezafungin is a novel echinocandin with unusual stability, solubility, and a long half-life. Phase 3 trials will provide the data on efficacy for the treatment of invasive candidiasis.	[123,124,125]
	Combination of Azoles such as fluconazole, voriconazaole and itraconazole, posaconazole and ravuconazole	As described earlier azoles inhibit lanosterol 14α demethylase, an enzyme for ergosterol biosynthesis. Causes limiting toxicity of the drug. Azoles revealed fungistatic activity against Candida spp	It is an FDA-approved drug to treat various kinds of invasive candidiasis. Study stated that resistance to fluconazole is most common in C.auris, C.glabrata and C.parapsilosis.	[3,19,126,127]
	Lipid formulation of Amphotericin B	Liposomal amphotericin B used as an antifungal therapy intolerance of other classes may favor the use of liposomal amphotericin B. Provided in combination with other antifungal medications. LAmB was successfully used to treat C. glabrata and C. auris infection because these species produced resistance with azole and echinocandin treatment.	In addition to being FDA-approved, amphotericin B works against a wider range of Candida species. ∙	[23,113]
	Flucytosine	Flucytosine was developed as an antimetabolite, it inhibits fungal protein synthesis, and inhibits fungal DNA synthesis through the inhibition of thymidylate synthetase. Hematological toxicity risk is associated with flucytosine administration, so the monitoring of blood cell count is necessary during treatment.	This drug has a synergistic effect with amphotericin B, and it is used as a combination therapy for candidiasis infection. The use of monotherapy was limited due to emerging resistance. ∙	[128,129]
Mucormycosis	Surgical Treatment	The European Confederation of Medical Mycology (ECMM) and Mycoses Study Group Education and Research Consortium state that complete surgical intervention should take place first for the most effective treatment. Surgery is recommended for rhino-orbito-cerebral infection and soft tissue infection. It may be helpful in the case of a single localized pulmonary lesion, but impossible in the case of disseminated mucormycosis.	An interventional, open-label study of endoscopic surgical treatment of rhino-sinus mucormycosis involve 11 patients. Outcome measures that it controls the survival rate in association with antifungal therapy. Local control was obtained at 1 month and survival rate at 3 months.	[3,130] NCT02226705
	First-line treatment involves the use of liposomal amphotericin B (LAMB) at doses between 5 and 10 mg/kg/day.	The only antifungal medication that is currently approved for the treatment of mucormycosis is amphotericin B deoxycholate (AmB). A high dose (10 mg/kg/day) causes impairment in renal functions as shown by a doubling of serum creatinine level noted in 40% of patients. Some patients reported electrolyte imbalance.	A prospective, uncontrolled French mycosis study reported that 12 weeks of treatment of LAMB 10 mg/kg/day in combination with surgery seems to be a good response rate, but causes renal damage as a side effect.	[47,61,130,131]
	Posaconazole is given orally (oral suspension), in a dose range of 200 mg, three to four times daily.	The drug becomes effective approximately after a fortnight. Posaconazole acts by depleting ergosterol from the fungal cells. To overcome oral suspension therapeutic failure, its pharmacokinetic, and for enhanced bioavailability, it was formulated in tablet or IV injections.	In November 2013, the FDA granted approval for an oral posaconazole tablet with delayed release and multiple pivotal Phase III trials served as the foundation for the FDA and EMA’s authorization of posaconazole. An interventional, randomized open-label phase 2 study was carried out on 98 patients, to determine the safety, efficacy, tolerance, and PK of posaconazole. ∙	[77,130,132] NCT00034671
	Isavuconazole in the dose range of 200 mg did on days 1–2 andthen 200 mg per day for 3–6 months.	Isavuconazole has been given FDA approval for the treatment of infectious diseases caused by mucormycosis No adverse side effects such as hepatotoxicity or nephrotoxicity were reported. Isavuconazole is available in oral and IV formulations and has many advantages such as linear kinetics, less drug interaction, less toxicity, and good oral bioavailability. None of the adverse effects such as hepatotoxicity, or QT prolongation had been reported for isavuconazole.	An observational study of isavuconazole with 600 participants to check effectiveness, safety, and utilization of retrospective chart in mucormycosis patients. Primary outcome measures the overall response, including clinical response, radiological, mycological response, and mortality. Additionally, in clinical trials, isavuconazole has shown similar potency compared to voriconazole.	[77,117,130,133] NCT04550936
	Combination Therapy: Clinical advantages of this approach include the drug’s synergistic effects and broader coverage of pathogens than monotherapy.	In a murine mucormycosis model, combination therapy using LAmB and isavuconazole results in a synergistic advancement. However, invasive zygomycosis has been successfully treated using a combination of oral TRB and AMB. Patients with rhino-orbital-cerebral mucormycosis who received caspofungin and amphotericin B together showed a prognostic value.	Clinical trials conducted in this setting show caspofungin monotherapy to have an overall success rate of 45% to 60%. In a study, it was concluded that both caspofungin and liposomal amphotericin B were equally safe when used in combination therapy.	[133,134,135,136]
	Terbinafine and itraconazole seem to be effective treatments for ROCM brought on by *R. oryzae* and *R. microsporus,* respectively.	In combination with amphotericin B and voriconazole, terbinafine exhibit additive impacts against the Rhizopus, Rhizomucor, and Mucor species.	This was an observational, single-center study. In which 322 patients having mucormycosis have been involved. Among them, anti-fungal susceptibility involves 150 patients, for itraconazole 97.7% of R.orzye had MIC < 2 µg/mL. However, 36.5% of R.microporus had MIC < 2 µg/mL. for terabinafine 85.2% of R.microporus has MIC <2 µg/ml	[64,137]
	Ibrexafungerp with a suggested dosage of 300 mg twice each for one day can be administered.	For the diagnosis and treatment of invasive candidiasis, mucormycosis, and invasive aspergillosis, the FDA has granted QIDP and fast-track designations for oral and IV formulations of ibrexafungerp.	Ibrexafungerp has recently received FDA approval for oral administration to postmenstrual pediatric and adult female VVC patients.	[138]

## Data Availability

Not applicable.

## Abbreviations

COVID-19	Coronavirus Disease 2019
SARS-CoV-2	Severe Acute Respiratory Syndrome- Coronavirus 2
HIV	Human immunodeficiency virus
RT‒PCR	Real-Time- Polymerase Chain Reaction
CT	Computed Tomography
CRISPR‒Cas 9	clustered regularly interspaced short palindromic repeats-CRISPR associated protein 9 (CRISPR‒Cas 9)
MALDI-TOF	matrix-assisted laser desorption ionization-time of flight
FISH	Fluorescence In Situ Hybridization
LAmB	Liposomal amphotericin B
FDA	Food and Drug Administrations
EMA	European Medicines Agency
TRB	Terbinafine
AMB	Amphotericin B deoxycholate
QIDP	Qualified Infectious Disease Product
VVC	Vulvovaginal candidiasis
CAM	COVID-19-associated mycosis
CAPA	COVID-19-associated pulmonary aspergillosis
CAC	COVID-19-associated Candida

## References

[1] Seyedjavadi S.S., Bagheri P., Nasiri M.J., Razzaghi-Abyaneh M., Goudarzi M. (2022). Fungal Infection in Co-Infected Patients With COVID-19: An Overview of Case Reports/Case Series and Systematic Review. Front. Microbiol..

[2] Sanyaolu A., Okorie C., Marinkovic A., Patidar R., Younis K., Desai P., Hosein Z., Padda I., Mangat J., Altaf M. (2020). Comorbidity and Its Impact on Patients with COVID-19. SN Compr. Clin. Med..

[3] Bhatt K., Agolli A., Patel M.H., Garimella R., Devi M., Garcia E., Amin H., Domingue C., Del Castillo R.G., Sanchez-Gonzalez M. (2021). High Mortality Co-Infections of COVID-19 Patients: Mucormycosis and Other Fungal Infections. Discoveries.

[4] Coskun A.S., Durmaz S.O. (2021). Fungal Infections in COVID-19 Intensive Care Patients. Pol. J. Microbiol..

[5] Hughes S., Troise O., Donaldson H., Mughal N., Moore L.S.P. (2020). Bacterial and Fungal Coinfection among Hospitalized Patients with COVID-19: A Retrospective Cohort Study in a UK Secondary-Care Setting. Clin. Microbiol. Infect..

[6] Senok A., Alfaresi M., Khansaheb H., Nassar R., Hachim M., Al Suwaidi H., Almansoori M., Alqaydi F., Afaneh Z., Mohamed A. (2021). Coinfections in Patients Hospitalized with COVID-19: A Descriptive Study from the United Arab Emirates. Infect. Drug Resist..

[7] Garcia-Vidal C., Sanjuan G., Moreno-García E., Puerta-Alcalde P., Garcia-Pouton N., Chumbita M., Fernandez-Pittol M., Pitart C., Inciarte A., Bodro M. (2021). Incidence of Co-Infections and Superinfections in Hospitalized Patients with COVID-19: A Retrospective Cohort Study. Clin. Microbiol. Infect..

[8] Chavda V.P., Apostolopoulos V. (2021). Mucormycosis—An Opportunistic Infection in the Aged Immunocompromised Individual: A Reason for Concern in COVID-19. Maturitas.

[9] Shishido A.A., Mathew M., Baddley J.W. (2022). Overview of COVID-19-Associated Invasive Fungal Infection. Curr Fungal Infect Rep.

[10] Kundu R., Singla N. (2022). COVID-19 and Plethora of Fungal Infections. Curr. Fungal Infect. Rep..

[11] Arastehfar A., Carvalho A., Hong Nguyen M., Hedayati M.T., Netea M.G., Perlin D.S., Hoenigl M. (2020). COVID-19-Associated Candidiasis (CAC): An Underestimated Complication in the Absence of Immunological Predispositions?. J. Fungi.

[12] Amin A., Vartanian A., Poladian N., Voloshko A., Yegiazaryan A., Al-Kassir A.L., Venketaraman V. (2021). Root Causes of Fungal Coinfections in COVID-19 Infected Patients. Infect. Dis. Rep..

[13] Singh A.K., Singh R., Joshi S.R., Misra A. (2021). Mucormycosis in COVID-19: A Systematic Review of Cases Reported Worldwide and in India. Diabetes Metab. Syndr..

[14] Chiurlo M., Mastrangelo A., Ripa M., Scarpellini P. (2021). Invasive Fungal Infections in Patients with COVID-19: A Review on Pathogenesis, Epidemiology, Clinical Features, Treatment, and Outcomes. New Microbiol..

[15] Mahalaxmi I., Jayaramayya K., Venkatesan D., Subramaniam M.D., Renu K., Vijayakumar P., Narayanasamy A., Gopalakrishnan A.V., Kumar N.S., Sivaprakash P. (2021). Mucormycosis: An Opportunistic Pathogen during COVID-19. Environ. Res..

[16] Hernández J.L., Buckley C.J. (2022). Mucormycosis.

[17] Zmeili O.S., Soubani A.O. (2007). Pulmonary Aspergillosis: A Clinical Update. QJM: Int. J. Med..

[18] Latgé J.P., Chamilos G. (2020). Aspergillus Fumigatus and Aspergillosis in 2019. Clin. Microbiol. Rev..

[19] Dagenais T.R.T., Keller N.P. (2009). Pathogenesis of Aspergillus Fumigatus in Invasive Aspergillosis. Clin. Microbiol. Rev..

[20] Panackal A.A., Bennett J.E., Williamson P.R. (2014). Treatment Options in Invasive Aspergillosis. Curr. Treat. Options Infect. Dis..

[21] Reichenberger F., Habicht J.M., Gratwohl A., Tamm M. (2002). Diagnosis and Treatment of Invasive Pulmonary Aspergillosis in Neutropenic Patients. Eur. Respir. J..

[22] Martínez-Jiménez S., Rosado-de-Christenson M.L., Carter B.W. (2017). Invasive Aspergillosis. Spec. Imaging HRCT Lung.

[23] Barantsevich N., Barantsevich E. (2022). Diagnosis and Treatment of Invasive Candidiasis. Antibiotics.

[24] Zarrin M., Mahmoudabadi A.Z. (2009). Invasive Candidiasis; A Review Article. Jundishapur J. Microbiol..

[25] Calandra T., Roberts J.A., Antonelli M., Bassetti M., Vincent J.L. (2016). Diagnosis and Management of Invasive Candidiasis in the ICU: An Updated Approach to an Old Enemy. Crit. Care.

[26] Corzo-Leon D.E., Peacock M., Rodriguez-Zulueta P., Salazar-Tamayo G.J., MacCallum D.M. (2021). General Hospital Outbreak of Invasive Candidiasis Due to Azole-Resistant Candida Parapsilosis Associated with an Erg11 Y132F Mutation. Med. Mycol..

[27] Van De Veerdonk F.L., Kullberg B.J., Netea M.G. (2010). Pathogenesis of Invasive Candidiasis. Curr. Opin. Crit. Care.

[28] Pappas P.G., Lionakis M.S., Arendrup M.C., Ostrosky-Zeichner L., Kullberg B.J. (2018). Invasive Candidiasis. Nat. Rev. Dis. Prim..

[29] Singh D.K., Tóth R., Gácser A. (2020). Mechanisms of Pathogenic Candida Species to Evade the Host Complement Attack. Front. Cell. Infect. Microbiol..

[30] Ahmed N., Mahmood M.S., Ullah M.A., Araf Y., Rahaman T.I., Moin A.T., Hosen M.J. (2022). COVID-19-Associated Candidiasis: Possible Patho-Mechanism, Predisposing Factors, and Prevention Strategies. Curr. Microbiol..

[31] Dekkers B.G.J., Veringa A., Marriott D.J.E., Boonstra J.M., van der Elst K.C.M., Doukas F.F., McLachlan A.J., Alffenaar J.W.C. (2018). Invasive Candidiasis in the Elderly: Considerations for Drug Therapy. Drugs Aging.

[32] Kayaaslan B., Eser F., Kaya Kalem A., Bilgic Z., Asilturk D., Hasanoglu I., Ayhan M., Tezer Tekce Y., Erdem D., Turan S. (2021). Characteristics of Candidemia in COVID-19 Patients; Increased Incidence, Earlier Occurrence and Higher Mortality Rates Compared to Non-COVID-19 Patients. Mycoses.

[33] Flevari A., Theodorakopoulou M., Velegraki A., Armaganidis A., Dimopoulos G. (2013). Treatment of Invasive Candidiasis in the Elderly: A Review. Clin. Interv. Aging.

[34] Spellberg B., Edwards J., Ibrahim A. (2005). Novel Perspectives on Mucormycosis: Pathophysiology, Presentation, and Management. Clin. Microbiol. Rev..

[35] Mucormycosis | Fungal Diseases | CDC. https://www.cdc.gov/fungal/diseases/mucormycosis/index.html.

[36] Shah N.N., Khan Z., Ahad H., Elderdery A.Y., Alomary M.N., Atwah B., Alhindi Z., Alsugoor M.H., Elkhalifa A.M.E., Nabi S. (2022). Mucormycosis an Added Burden to Covid-19 Patients: An in-Depth Systematic Review. J. Infect. Public Health.

[37] Koehler P., Cornely O.A., Böttiger B.W., Dusse F., Eichenauer D.A., Fuchs F., Hallek M., Jung N., Klein F., Persigehl T. (2020). COVID-19 Associated Pulmonary Aspergillosis. Mycoses.

[38] Song G., Liang G., Liu W. (2020). Fungal Co-Infections Associated with Global COVID-19 Pandemic: A Clinical and Diagnostic Perspective from China. Mycopathologia.

[39] Ibrahim A.S., Spellberg B., Walsh T.J., Kontoyiannis D.P. (2012). Pathogenesis of Mucormycosis. Clin. Infect. Dis..

[40] Farghly Youssif S., Abdelrady M.M., Thabet A.A., Abdelhamed M.A., Gad M.O.A., Abu-Elfatth A.M., Saied G.M., Goda I., Algammal A.M., Batiha G.E.S. (2022). COVID-19 Associated Mucormycosis in Assiut University Hospitals: A Multidisciplinary Dilemma. Sci. Rep..

[41] (2021). Centers for Disease Control and Prevention; National Center for Emerging and Zoonotic Infectious Diseases (NCEZID); Division of Foodborne, Waterborne, and Environmental Diseases (DFWED). Fungal Disease- Mucormycosis. *Centers for Disease Control and Prevention*. https://www.cdc.gov/fungal/diseases/mucormycosis/.

[42] Castrejón-Pérez A.D., Welsh E.C., Miranda I., Ocampo-Candiani J., Welsh O. (2017). Cutaneous Mucormycosis. An. Bras. Dermatol..

[43] Sharma A., Bano G., Malik A. (2021). Mucormycosis: A Manifestation in COVID-19 Infection. Indian J. Pharm. Pharmacol..

[44] Petrikkos G., Skiada A., Lortholary O., Roilides E., Walsh T.J., Kontoyiannis D.P. (2012). Epidemiology and Clinical Manifestations of Mucormycosis. Clin. Infect. Dis..

[45] Verma G., Lobo D., Walker R., Bose S., Gupta K. (1995). Disseminated Mucormycosis in Healthy Adults. J. Postgrad. Med..

[46] Alekseyev K., Didenko L., Chaudhry B. (2021). Rhinocerebral Mucormycosis and COVID-19 Pneumonia. J. Med. Cases.

[47] Spellberg B. (2012). Gastrointestinal Mucormycosis: An Evolving Disease. Gastroenterol. Hepatol..

[48] Kaur J., Singh U., Pradhan U., Singh G., Agarwal P.N. (2021). A Rare Case of Gastrointestinal Mucormycosis. Cureus.

[49] Agrawal R., Yeldandi A., Savas H., Parekh N.D., Lombardi P.J., Hart E.M. (2020). Pulmonary Mucormycosis: Risk Factors, Radiologic Findings, and Pathologic Correlation. Radiographics.

[50] Fernandez J.F., Maselli D.J., Simpson T., Restrepo M.I. (2013). Pulmonary Mucormycosis: What Is the Best Strategy for Therapy?. Respir. Care.

[51] De Macedo P.M., Freitas A.D., Bártholo T.P., Bernardes-Engemann A.R., Almeida M., de A. (2021). Almeida-Silva, F.; Zancopé-Oliveira, R.M.; Almeida-Paes, R. Acute Pulmonary Histoplasmosis Following COVID-19: Novel Laboratorial Methods Aiding Diagnosis. J. Fungi.

[52] Gerber V., Ruch Y., Chamaraux-Tran T.N., Oulehri W., Schneider F., Lindner V., Greigert V., Denis J., Brunet J., Danion F. (2021). Detection of Pneumocystis Jirovecii in Patients with Severe COVID-19: Diagnostic and Therapeutic Challenges. J. Fungi.

[53] Regalla D., VanNatta M., Alam M., Malek A.E. (2022). COVID-19-Associated Cryptococcus Infection (CACI): A Review of Literature and Clinical Pearls. Infection.

[54] Ghanem H., Sivasubramanian G. (2021). Cryptococcus Neoformans Meningoencephalitis in an Immunocompetent Patient after COVID-19 Infection. Case Rep. Infect. Dis..

[55] Perez Del Nogal G., Mata A., Ernest P., Salinas I. (2022). Disseminated Histoplasmosis in an Immunocompetent Patient with COVID-19 Pneumonia. BMJ Case Rep. CP.

[56] Khanna A., Sinha A., Kumar P., Pandey K.K. (2022). Acute Localized Pulmonary Histoplasmosis -Another Bug out of COVID’s Pandora Box!. Lung India.

[57] Paul M., Sasidharan J., Taneja J., Chatterjee K., Abbas S.Z., Chowdhury V., Das A. (2022). Invasive Mucormycosis and Aspergillosis Coinfection Associated with Post-COVID-19 Pneumonia in a Tertiary Care Hospital. Med. Mycol. J..

[58] COVID-19-Associated Subacute Invasive Pulmonary Aspergillosis—Swain—2022—Mycoses—Wiley Online Library. https://onlinelibrary.wiley.com/doi/abs/10.1111/myc.13369.

[59] Salas B., McCullagh I., Cranfield K., Fagan C., Geering A., Robb A. (2022). COVID-19-Associated Pulmonary Aspergillosis: A Year-Long Retrospective Case Series. COVID.

[60] Vitale R.G., Afeltra J., Seyedmousavi S., Giudicessi S.L., Romero S.M. (2022). An Overview of COVID-19 Related to Fungal Infections: What Do We Know after the First Year of Pandemic?. Braz. J. Microbiol..

[61] Hoenigl M., Seidel D., Sprute R., Cunha C., Oliverio M., Goldman G.H., Ibrahim A.S., Carvalho A. (2022). COVID-19-Associated Fungal Infections. Nat. Microbiol..

[62] Garre V. (2022). Recent Advances and Future Directions in the Understanding of Mucormycosis. Front. Cell. Infect. Microbiol..

[63] Al-Tawfiq J.A., Alhumaid S., Alshukairi A.N., Temsah M.-H., Barry M., Al Mutair A., Rabaan A.A., Al-Omari A., Tirupathi R., AlQahtani M. (2021). COVID-19 and Mucormycosis Superinfection: The Perfect Storm. Infection.

[64] Madhavan Y., Sai K.V., Shanmugam D.K., Manimaran A., Guruviah K., Mohanta Y.K., Venugopal D.C., Mohanta T.K., Sharma N., Muthupandian S. (2022). Current Treatment Options for COVID-19 Associated Mucormycosis: Present Status and Future Perspectives. J. Clin. Med..

[65] Szydłowicz M., Matos O. (2021). Pneumocystis Pneumonia in the COVID-19 Pandemic Era: Similarities and Challenges. Trends Parasitol..

[66] Moradians V., Shateri Amiri B., Bahadorizadeh L., Gholizadeh Mesgarha M., Sadeghi S. (2022). Concurrent COVID-19 and Pneumocystis Carinii Pneumonia in a Patient Subsequently Found to Have Underlying Hairy Cell Leukemia. Radiol. Case Rep..

[67] Jeican I.I., Inișca P., Gheban D., Tăbăran F., Aluaș M., Trombitas V., Cristea V., Crivii C., Junie L.M., Albu S. (2021). COVID-19 and Pneumocystis Jirovecii Pulmonary Coinfection—The First Case Confirmed through Autopsy. Medicina.

[68] Chastain D.B., Henao-Martínez A.F., Dykes A.C., Steele G.M., Stoudenmire L.L., Thomas G.M., Kung V., Franco-Paredes C. (2022). Missed Opportunities to Identify Cryptococcosis in COVID-19 Patients: A Case Report and Literature Review. Ther. Adv. Infect. Dis..

[69] Chastain D.B., Kung V.M., Golpayegany S., Jackson B.T., Franco-Paredes C., Barahona L.V., Thompson III G.R., Henao-Martínez A.F. (2022). Cryptococcosis among Hospitalised Patients with COVID-19: A Multicentre Research Network Study. Mycoses.

[70] Choi H.S. (2022). Pulmonary Cryptococcosis after Recovery from COVID-19 in an Immunocompetent Patient: A Rare Case Report. Medicine.

[71] Maldonado I., Elisiri M.E., Fernández-Canigia L., Sánchez A.V., López L., Toranzo A.I., López-Joffre C., González-Fraga S., Canteros C.E. (2022). COVID-19 Associated with Disseminated Histoplasmosis in a Kidney Transplant Patient. Rev. Argent. Microbiol..

[72] Munipati S., Rachamadugu H., Avileli S., Avula R., Beladona N.J., Boyapally S.R. (2022). Microbiological Profile of Post-COVID-19 Mucormycosis in Various Samples. Int. J. Sci. Res. Dent. Med. Sci..

[73] Machado M., Valerio M., Álvarez-Uría A., Olmedo M., Veintimilla C., Padilla B., De la Villa S., Guinea J., Escribano P., Ruiz-Serrano M.J. (2021). Invasive Pulmonary Aspergillosis in the COVID-19 Era: An Expected New Entity. Mycoses.

[74] Fungal Diseases and COVID-19|CDC. https://www.cdc.gov/fungal/covid-fungal.html.

[75] Zia M., Goli M. (2021). Predisposing Factors of Important Invasive Fungal Coinfections in COVID-19 Patients: A Review Article. J. Int. Med. Res..

[76] Soni S., Namdeo Pudake R., Jain U., Chauhan N. (2022). A Systematic Review on SARS-CoV-2-associated Fungal Coinfections. J. Med. Virol..

[77] Bharadwaj R., Thilagavathy S. (2021). Mucormycosis in COVID-19: A Clinico-Microbiological Dilemma. Kauverian Sci. J..

[78] Gasmi A., Noor S., Tippairote T., Dadar M., Menzel A., Bjørklund G. (2020). Individual Risk Management Strategy and Potential Therapeutic Options for the COVID-19 Pandemic. Clin. Immunol..

[79] Mani J.S., Johnson J.B., Steel J.C., Broszczak D.A., Neilsen P.M., Walsh K.B., Naiker M. (2020). Natural Product-Derived Phytochemicals as Potential Agents against Coronaviruses: A Review. Virus Res..

[80] De Almeida Brasiel P.G. (2020). The Key Role of Zinc in Elderly Immunity: A Possible Approach in the COVID-19 Crisis. Clin. Nutr. Espen.

[81] Bermano G., Méplan C., Mercer D.K., Hesketh J.E. (2021). Selenium and Viral Infection: Are There Lessons for COVID-19?. Br. J. Nutr..

[82] Sio S., De Buomprisco G., La Torre G., Lapteva E., Perri R., Greco E., Mucci N., Cedrone F. (2020). The Impact of COVID-19 on Doctors’ Well-Being: Results of a Web Survey during the Lockdown in Italy. Eur. Rev. Med. Pharmacol. Sci..

[83] Anca P.S., Toth P.P., Kempler P., Rizzo M. (2021). Gender Differences in the Battle against COVID-19: Impact of Genetics, Comorbidities, Inflammation and Lifestyle on Differences in Outcomes. Int. J. Clin. Pract..

[84] Gasmi A., Chirumbolo S., Peana M., Noor S., Menzel A., Dadar M., Bjørklund G. (2022). The Role of Diet and Supplementation of Natural Products in COVID-19 Prevention. Biol. Trace Elem. Res..

[85] Xiao T., Mu T., Shen S., Song Y., Yang S., He J. (2022). A Dynamic Physical-Distancing Model to Evaluate Spatial Measures for Prevention of Covid-19 Spread. Phys. A Stat. Mech. Its Appl..

[86] Pan L., Wang J., Wang X., Ji J.S., Ye D., Shen J., Li L., Liu H., Zhang L., Shi X. (2022). Prevention and Control of Coronavirus Disease 2019 (COVID-19) in Public Places. Environ. Pollut..

[87] Tripathi M.K., Singh P., Sharma S., Singh T.P., Ethayathulla A.S., Kaur P. (2020). Identification of Bioactive Molecule from Withania Somnifera (Ashwagandha) as SARS-CoV-2 Main Protease Inhibitor. J. Biomol. Struct. Dyn..

[88] Jayawardena R., Sooriyaarachchi P., Chourdakis M., Jeewandara C., Ranasinghe P. (2020). Enhancing Immunity in Viral Infections, with Special Emphasis on COVID-19: A Review. Diabetes Metab. Syndr..

[89] Gasmi A., Tippairote T., Mujawdiya P.K., Peana M., Menzel A., Dadar M., Benahmed A.G., Bjørklund G. (2021). The Microbiota-Mediated Dietary and Nutritional Interventions for COVID-19. Clin. Immunol..

[90] Barazzoni R., Bischoff S.C., Breda J., Wickramasinghe K., Krznaric Z., Nitzan D., Pirlich M., Singer P. (2020). ESPEN Expert Statements and Practical Guidance for Nutritional Management of Individuals with SARS-CoV-2 Infection. Clin. Nutr..

[91] Shin B., Koh W.J., Jeong B.H., Yoo H., Park H.Y., Suh G.Y., Kwon O.J., Jeon K. (2014). Serum Galactomannan Antigen Test for the Diagnosis of Chronic Pulmonary Aspergillosis. J. Infect..

[92] Arvanitis M., Anagnostou T., Fuchs B.B., Caliendo A.M., Mylonakis E. (2014). Molecular and Nonmolecular Diagnostic Methods for Invasive Fungal Infections. Clin. Microbiol. Rev..

[93] Badiee P., Hashemizadeh Z. (2014). Opportunistic Invasive Fungal Infections: Diagnosis & Clinical Management. Indian J. Med. Res..

[94] Azhar A., Khan W.H., Khan P.A., Alhosaini K., Owais M., Ahmad A. (2022). Mucormycosis and COVID-19 Pandemic: Clinical and Diagnostic Approach. J. Infect. Public Health.

[95] Kozel T.R., Wickes B. (2014). Fungal Diagnostics. Cold Spring Harb. Perspect. Med..

[96] Rickerts V., Mousset S., Lambrecht E., Tintelnot K., Schwerdtleger R., Presterl E., Jacobi V., Just-Nübling G., Bialek R. (2007). Comparison of Histopathological Analysis, Culture, and Polymerase Chain Reaction Assays to Detect Invasive Mold Infections from Biopsy Specimens. Clin. Infect. Dis..

[97] Liu G., Rusling J.F. (2021). COVID-19 Antibody Tests and Their Limitations. ACS Sens..

[98] Singhal N., Kumar M., Kanaujia P.K., Virdi J.S. (2015). MALDI-TOF Mass Spectrometry: An Emerging Technology for Microbial Identification and Diagnosis. Front. Microbiol..

[99] Chavda V.P., Patel A.B., Pandya A., Vora L.K., Patravale V., Tambuwala Z.M., Aljabali A.A.A., Serrano-Aroca Á., Mishra V., Tambuwala M.M. (2022). Co-Infection Associated with SARS-CoV-2 and Their Management. Futur. Sci. OA.

[100] Rahimi H., Salehiabar M., Barsbay M., Ghaffarlou M., Kavetskyy T., Sharafi A., Davaran S., Chauhan S.C., Danafar H., Kaboli S. (2021). CRISPR Systems for COVID-19 Diagnosis. ACS Sens..

[101] Theel E.S., Doern C.D. (2013). β-d-Glucan Testing Is Important for Diagnosis of Invasive Fungal Infections. J. Clin. Microbiol..

[102] Azar M.M., Hage C.A. (2017). Laboratory Diagnostics for Histoplasmosis. J. Clin. Microbiol..

[103] De Heer K., Gerritsen M.G., Visser C.E., Leeflang M.M. (2019). Galactomannan Detection in Broncho-alveolar Lavage Fluid for Invasive Aspergillosis in Immunocompromised Patients. Cochrane Database Syst. Rev..

[104] Guo Y.-L., Chen Y.-Q., Wang K., Qin S.-M., Wu C., Kong J.-L. (2010). Accuracy of BAL Galactomannan in Diagnosing Invasive Aspergillosis: A Bivariate Metaanalysis and Systematic Review. CHEST.

[105] Silva L.N., de Mello T.P., de Souza Ramos L., Branquinha M.H., Roudbary M., dos Santos A.L.S. (2020). Fungal Infections in COVID-19-Positive Patients: A Lack of Optimal Treatment Options. Curr. Top. Med. Chem..

[106] Treatment for Aspergillosis|Aspergillosis|Types of Fungal Diseases|Fungal Diseases|CDC. https://www.cdc.gov/fungal/diseases/aspergillosis/treatment.html.

[107] Ong V., Hough G., Schlosser M., Bartizal K., Balkovec J.M., James K.D., Krishnan B.R. (2016). Preclinical Evaluation of the Stability, Safety, and Efficacy of CD101, a Novel Echinocandin. Antimicrob. Agents Chemother..

[108] Treatment|Invasive Candidiasis|Candidiasis|Types of Diseases|Fungal Diseases|CDC. https://www.cdc.gov/fungal/diseases/candidiasis/invasive/treatment.html.

[109] Menon A.A., Berg D.D., Brea E.J., Deutsch A.J., Kidia K.K., Thurber E.G., Polsky S.B., Yeh T., Duskin J.A., Holliday A.M. (2020). A Case of COVID-19 and Pneumocystis Jirovecii Coinfection. Am. J. Respir. Crit. Care Med..

[110] Goldstein E.J.C., Spellberg B., Walsh T.J., Kontoyiannis D.P., Edwards J., Ibrahim A.S. (2009). Recent Advances in the Management of Mucormycosis: From Bench to Bedside. Clin. Infect. Dis..

[111] Ellsworth M., Ostrosky-Zeichner L. (2020). Isavuconazole: Mechanism of Action, Clinical Efficacy, and Resistance. J. Fungi.

[112] Jones C.T., Kopf R.S., Tushla L., Tran S., Hamilton C., Lyman M., McMullen R., Shah D., Stroman A., Wilkinson E. (2022). A Care Step Pathway for the Diagnosis and Treatment of COVID-19-Associated Invasive Fungal Infections in the Intensive Care Unit. Crit. Care Nurse.

[113] Domán M., Bányai K. (2022). COVID-19-Associated Fungal Infections: An Urgent Need for Alternative Therapeutic Approach?. Front. Microbiol..

[114] Groll A.H., Gastine S., Hempel G. (2020). Chapter 9—Therapeutic Drug Monitoring for Antifungal Triazoles: Pharmacologic Background and Current Status. Methods of Therapeutic Drug Monitoring Including Pharmacogenetics.

[115] Jenks J.D., Hoenigl M. (2018). Treatment of Aspergillosis. J. Fungi.

[116] Shirley M., Scott L.J. (2016). Isavuconazole: A Review in Invasive Aspergillosis and Mucormycosis. Drugs.

[117] Jenks J.D., Salzer H.J., Prattes J., Krause R., Buchheidt D., Hoenigl M. (2018). Spotlight on Isavuconazole in the Treatment of Invasive Aspergillosis and Mucormycosis: Design, Development, and Place in Therapy. Drug Des. Dev. Ther..

[118] Greer N.D. (2007). Posaconazole (Noxafil): A New Triazole Antifungal Agent. Baylor University Medical Center Proceedings.

[119] Alexander B.D., Perfect J.R., Daly J.S., Restrepo A., Tobón A.M., Patino H., Hardalo C.J., Graybill J.R. (2008). Posaconazole as Salvage Therapy in Patients with Invasive Fungal Infections After Solid Organ Transplant. Transplantation.

[120] Walsh T.J., Raad I., Patterson T.F., Chandrasekar P., Donowitz G.R., Graybill R., Greene R.E., Hachem R., Hadley S., Herbrecht R. (2007). Treatment of Invasive Aspergillosis with Posaconazole in Patients Who Are Refractory to or Intolerant of Conventional Therapy: An Externally Controlled Trial. Clin. Infect. Dis..

[121] Liposomal Amphotericin B (AmBisome®) Efficacy in Confirmed Invasive Aspergillosis and Other Filamentous Fungal Infections in Immunocompromised Hosts: A Pooled Analysis—Cordonnier—2007—Mycoses—Wiley Online Library. https://onlinelibrary.wiley.com/doi/10.1111/j.1439-0507.2007.01362.x.

[122] Reichert-Lima F., Lyra L., Pontes L., Moretti M.L., Pham C.D., Lockhart S.R., Schreiber A.Z. (2018). Surveillance for Azoles Resistance in *Aspergillus* spp. Highlights a High Number of Amphotericin B-Resistant Isolates. Mycoses.

[123] Posteraro B., Torelli R., Vella A., Leone P.M., De Angelis G., De Carolis E., Ventura G., Sanguinetti M., Fantoni M. (2020). Pan-Echinocandin-Resistant Candida Glabrata Bloodstream Infection Complicating COVID-19: A Fatal Case Report. J. Fungi.

[124] Ben-Ami R. (2018). Treatment of Invasive Candidiasis: A Narrative Review. J. Fungi.

[125] Ham Y.Y., Lewis J.S., Thompson G.R. (2021). Rezafungin: A Novel Antifungal for the Treatment of Invasive Candidiasis. Future Microbiol..

[126] Govindarajan A., Bistas K.G., Ingold C.J., Aboeed A. (2022). Fluconazole. Kucers the Use of Antibiotics: A Clinical Review of Antibacterial, Antifungal, Antiparasitic, and Antiviral Drugs.

[127] Berkow E.L., Lockhart S.R. (2017). Fluconazole Resistance in *Candida* Species: A Current Perspective. IDR.

[128] Delma F.Z., Al-Hatmi A.M.S., Brüggemann R.J.M., Melchers W.J.G., de Hoog S., Verweij P.E., Buil J.B. (2021). Molecular Mechanisms of 5-Fluorocytosine Resistance in Yeasts and Filamentous Fungi. J. Fungi.

[129] Vermes A., Sijs H., van der Guchelaar H.-J. (2000). Flucytosine: Correlation between Toxicity and Pharmacokinetic Parameters. CHE.

[130] Subramaniyan V., Fuloria S., Darnal H.K., Meenakshi D.U., Sekar M., Nordin R., Bin Chakravarthi S., Sathasivam K.V., Khan S.A., Wu Y.S. (2021). COVID-19-Associated Mucormycosis and Treatments. Asian Pac. J. Trop. Med..

[131] Lanternier F., Poiree S., Elie C., Garcia-Hermoso D., Bakouboula P., Sitbon K., Herbrecht R., Wolff M., Ribaud P., Lortholary O. (2015). Prospective Pilot Study of High-Dose (10 Mg/Kg/Day) Liposomal Amphotericin B (L-AMB) for the Initial Treatment of Mucormycosis. J. Antimicrob. Chemother..

[132] Moore J.N., Healy J.R., Kraft W.K. (2015). Pharmacologic and Clinical Evaluation of Posaconazole. Expert Rev. Clin. Pharmacol..

[133] Rybak J.M., Marx K.R., Nishimoto A.T., Rogers P.D. (2015). Isavuconazole: Pharmacology, Pharmacodynamics, and Current Clinical Experience with a New Triazole Antifungal Agent. Pharmacother. J. Hum. Pharmacol. Drug Ther..

[134] Smith C., Lee S.C. (2022). Current Treatments against Mucormycosis and Future Directions. PLOS Pathog..

[135] Skiada A., Lass-Floerl C., Klimko N., Ibrahim A., Roilides E., Petrikkos G. (2018). Challenges in the Diagnosis and Treatment of Mucormycosis. Med. Mycol..

[136] Maertens J., Egerer G., Shin W.S., Reichert D., Stek M., Chandwani S., Shivaprakash M., Viscoli C. (2010). Caspofungin Use in Daily Clinical Practice for Treatment of Invasive Aspergillosis: Results of a Prospective Observational Registry. BMC Infect. Dis..

[137] Gupta P., Malhotra H.S., Saxena P., Singh R., Shukla D., Hasan M.S., Verma V., Banerjee G., Puri B., Dandu H. (2022). Utility of Itraconazole and Terbinafine in Mucormycosis: A Proof-of-Concept Analysis. J. Investig. Med..

[138] Hoenigl M., Sprute R., Egger M., Arastehfar A., Cornely O.A., Krause R., Lass-Flörl C., Prattes J., Spec A., Thompson G.R. (2021). The Antifungal Pipeline: Fosmanogepix, Ibrexafungerp, Olorofim, Opelconazole, and Rezafungin. Drugs.

